# Controlling recurrent scabies outbreaks in a geriatric hospital: impact of a prevention bundle and lessons from a breakthrough case

**DOI:** 10.3389/fpubh.2026.1807182

**Published:** 2026-04-07

**Authors:** Lanchuan Li, Renhua Li, Yifei Wang, Keli Qian

**Affiliations:** 1 Department of Infection Control, The Thirteenth People's Hospital of Chongqing, Chongqing, China; 2 Department of Infection Control, The First Affiliated Hospital of Chongqing Medical University, Chongqing, China

**Keywords:** caregiver management, comprehensive intervention, geriatric hospital, nosocomial transmission, scabies

## Abstract

**Background:**

Scabies represents a significant infection control challenge in geriatric care facilities. Traditional reactive strategies, such as isolating identified cases, have frequently proven inadequate in preventing recurrent outbreaks, underscoring the need for proactive, systematic approaches. This study aimed to evaluate the effectiveness of a comprehensive, proactive intervention strategy in controlling nosocomial scabies transmission in a large specialized geriatric hospital.

**Method:**

A before and after study was conducted, comparing a pre-intervention period (January 2021–December 2024) with an intervention period (January–December 2025). The multimodal strategy included mandatory admission screening and transfer isolation for all new inpatients, weekly active symptom surveillance of caregivers, and strict implementation of a “one patient, one caregiver” model coupled with infection prevention training.

**Results:**

Pre-intervention, 114 scabies cases were recorded, including 62 definite nosocomial transmissions and 18 infected caregivers. Post-intervention, 13 imported cases were intercepted at admission. Only one nosocomial transmission event (involving a nurse) was recorded, and no caregiver infections occurred. The reduction in nosocomial transmission was statistically significant (*P* < 0.001).

**Conclusion:**

A proactive, multimodal intervention bundle effectively interrupted nosocomial scabies transmission in a high-risk geriatric setting. This strategy should be considered for integration into standard infection control protocols in long-term care facilities.

## Introduction

Scabies, caused by the mite Sarcoptes scabiei var. hominis, is a highly contagious parasitic infestation. With an estimated global incidence exceeding 200 million cases annually and its classification as a neglected tropical disease by the World Health Organization in 2017, it poses a substantial public health burden, particularly in congregate settings (1–3). Transmission primarily occurs via direct skin-to-skin contact, while indirect spread can also take place through fomites contaminated with the mites, including bedding, clothing and towels used by infected individuals. The risk of scabies outbreaks rises dramatically in communal settings such as nursing homes, long-term care facilities and geriatric hospitals, where unchecked transmission chains may rapidly emerge (4, 5).

Older adult(s) patients, who typically exhibit impaired immune function and multiple comorbidities, are a high-risk group for severe scabies. Following infection, they commonly suffer from intractable pruritus, which not only elevates the susceptibility to secondary bacterial infections but also exerts a profound negative impact on their quality of life and mental health (6).

This study was conducted at a large-scale specialized geriatric hospital. The majority of inpatients are transferred from local older adult(s) care facilities, which are recognized as potential scabies reservoirs. Moreover, ward care relies primarily on caregivers, and the historical practice of assigning a single caregiver to attend to multiple patients concurrently has established an optimal route for scabies cross-transmission via caregiver-mediated contact (7). Despite the implementation of isolation interventions immediately after the identification of scabies cases between 2021 and 2024, recurrent nosocomial scabies outbreaks occurred consistently every summer during this period. This observation underscores the inherent limitations of the hospital's prior passive response strategies.

Against this backdrop, the Infection Control Department of our hospital implemented a prospective, multimodal comprehensive intervention strategy in 2025. This study aimed to evaluate the impact of a novel, multimodal intervention bundle on the incidence of nosocomial scabies transmission and to provide an evidence-based framework for infection control in similar geriatric care environments.

## Methods

### Study design

A before–after comparative study was conducted across all inpatient wards (600 beds) of a specialist geriatric hospital. The control period was January 2021 to December 2024, and the intervention period was January to December 2025. Ethical approval was waived as the study evaluated the implementation of routine infection control measures.

### Baseline infection control practices (2021–2024)

During the pre-intervention period, standard precautions including hand hygiene, gloves, and masks were routinely implemented. Confirmed scabies cases were isolated in single rooms, and contact precautions were applied. However, no systematic admission screening for scabies was performed, and dermatology consultation was only sought upon clinical suspicion. Caregivers, who were primarily employed by external agencies, often attended multiple patients simultaneously without formal infection prevention training or regular health surveillance. No restrictions were placed on caregiver–patient assignments.

### Intervention measures (2025)

**Figure 1:**
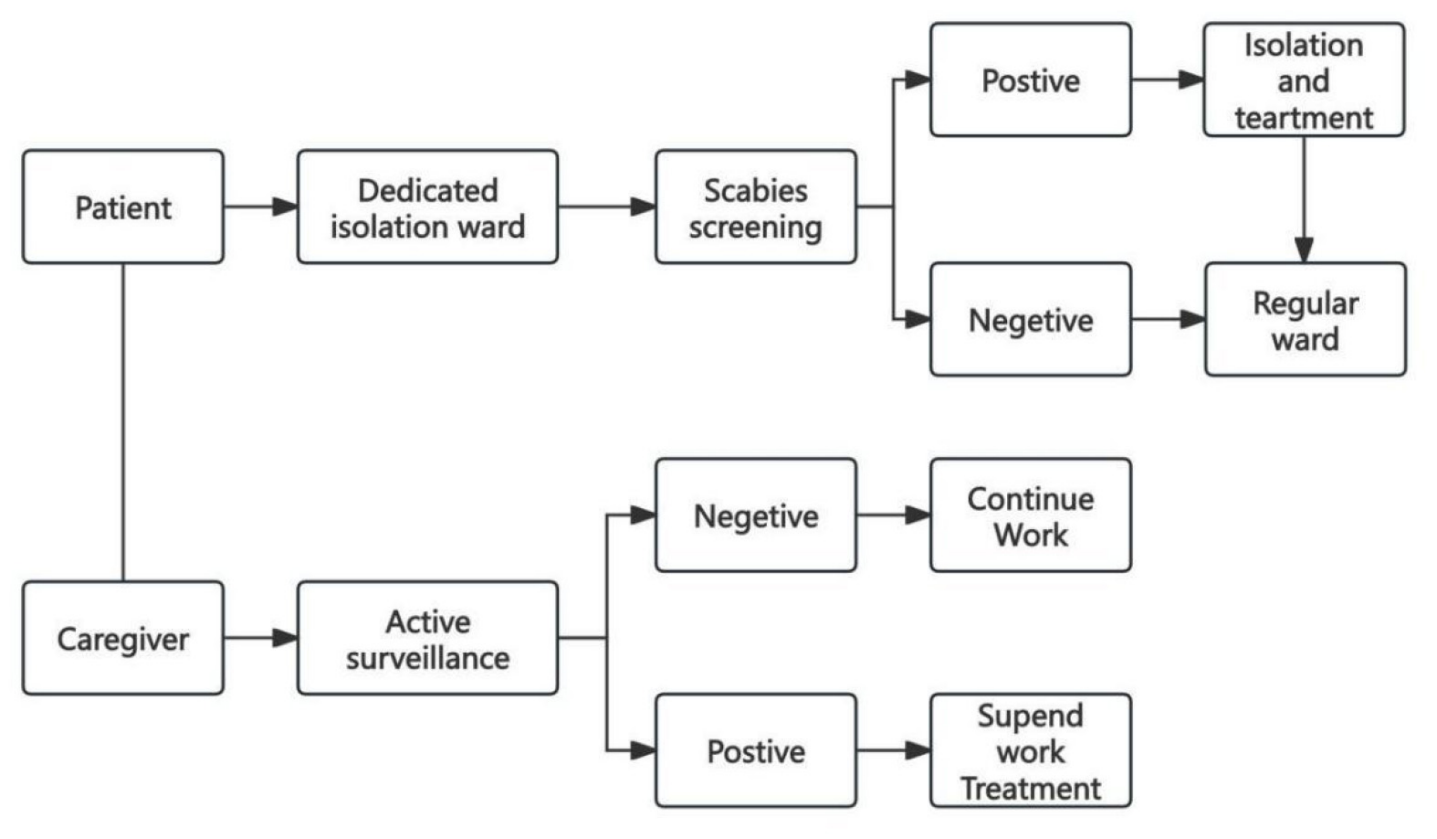
Flowchart of the proactive scabies prevention bundle implemented for patients and caregivers. The diagram outlines the two parallel pathways of the intervention. For patients, all new admissions undergo mandatory screening in a dedicated isolation ward; those with negative results are transferred to regular wards, while positive cases receive isolation and treatment. For caregivers, active weekly surveillance is performed; symptomatic individuals are suspended from work and treated, while asymptomatic caregivers continue work under continuous monitoring.

Starting from January 1, 2025, our hospital has established a multidisciplinary collaboration team consisting of the Medical Affairs Department, the Nursing Department, the Infection Prevention and Control Department, and the Dermatology Department. These three core intervention measures were implemented as mandatory protocol (Figure 1). Staff members from the Infection Control Department supervise the implementation of these measures.

First, admission screening and transfer isolation. All new admissions are initially placed in a single-bed isolation room with a private bathroom within their assigned ward, as no centralized isolation ward exists. Within 24 h, a dermatologist (at least attending physician level, rotating monthly) performs a full-body skin examination, focusing on typical sites. Patients with negative screening are transferred to regular wards; those with suspected or confirmed scabies remain in the isolation room for treatment and are managed under contact precautions. All medical staff in the isolation ward take contact isolation measures, especially when in close proximity, they need to wear disposable isolation gowns, surgical masks, and disposable gloves. After completing their work, strict hand hygiene disinfection must be performed.

Second, active surveillance of caregivers. The number of caregivers was increased from 30–40 to 50–60. The Nursing Department has established a health monitoring file for the caregivers. All caregivers undergo a baseline skin examination upon hiring. During employment, they are screened weekly by a rotating dermatologist for scabies symptoms. In addition, the caregiver management company conducts daily spot checks, and caregivers are required to self-report any skin abnormalities. If scabies is suspected, the caregiver is immediately suspended from work and referred for diagnosis and treatment; replacement is arranged by the management company from a trained reserve pool.

Adherence to infection prevention measures (e.g., hand hygiene, use of long-sleeved work clothes and isolation gowns) is monitored weekly by the Infection Control Department and Nursing Department, and daily by ward-based infection control nurses. If caring for or coming into contact with patients diagnosed with or suspected of having scabies, hand hygiene should be performed by washing hands with soap to achieve the complete elimination of scabies mites.

Third, optimization of the caregiver-patient care model. Strictly implement the “one patient, one caregiver” system. The Nursing Department uses the shift scheduling system to solidify the corresponding relationship between caregivers and patients, completely eliminating the phenomenon of one caregiver providing care for multiple patients simultaneously. At the same time, staff from the Infection Prevention and Control Department conduct infection prevention training for caregivers every quarter, emphasizing personal protection including long-sleeved work clothes, isolation gowns, and hand hygiene, etc.

### Data collection and evaluation indicators

Data were retrospectively collected from the hospital infection real-time monitoring system and the medical record system for the period 2021 to 2024, and prospectively collected for 2025. The dataset included the annual number of inpatients, the number of patients diagnosed with scabies, the number of nosocomial scabies cases, and the number of infected caregivers and nurses. Nosocomial scabies cases were defined as new cases occurring in patients who had no signs or symptoms of scabies at admission and developed symptoms ≥7 days after admission, with a confirmed dermatological diagnosis (microscopic identification of Sarcoptes scabiei or clinical diagnosis by a dermatologist) and a clear epidemiological link to a confirmed case during the incubation period (up to 6 weeks before symptom onset). Imported cases were those diagnosed within 48 h of admission, with no prior hospital exposure. The primary evaluation indicator was the number of in-hospital transmission event of scabies (defined as new cases of scabies that occurred during hospitalization and with clear epidemiological associations), and the secondary evaluation indicators were the total number of scabies cases per year and the number of cases of infection among caregivers.

### Statistical analysis

Data analysis was conducted using SPSS 22.0 software. Count data were expressed as frequencies (percentages). The χ^2^ test was used to compare the differences in the primary and secondary outcome indicators before and after the intervention (with the combined data from 2021 to 2024 as the pre-intervention period and 2025 as the post-intervention period). A *P* value < 0.05 was considered to indicate statistically significant differences.

## Result

### Comparison of effects before and after intervention

From 2021 to 2024, our hospital documented a total of 114 scabies cases, corresponding to an annual average of 28.5 cases, with an incidence proportion (risk) of 2.42 per 1,000 patients. During this four-year period, 62 definite episodes of nosocomial scabies transmission were confirmed, with a mean of 15.5 cases per year (1.32 per 1,000 patients); additionally, a cumulative total of 18 caregivers were affected (0.38 per 1,000 patients).

**Figure 2:**
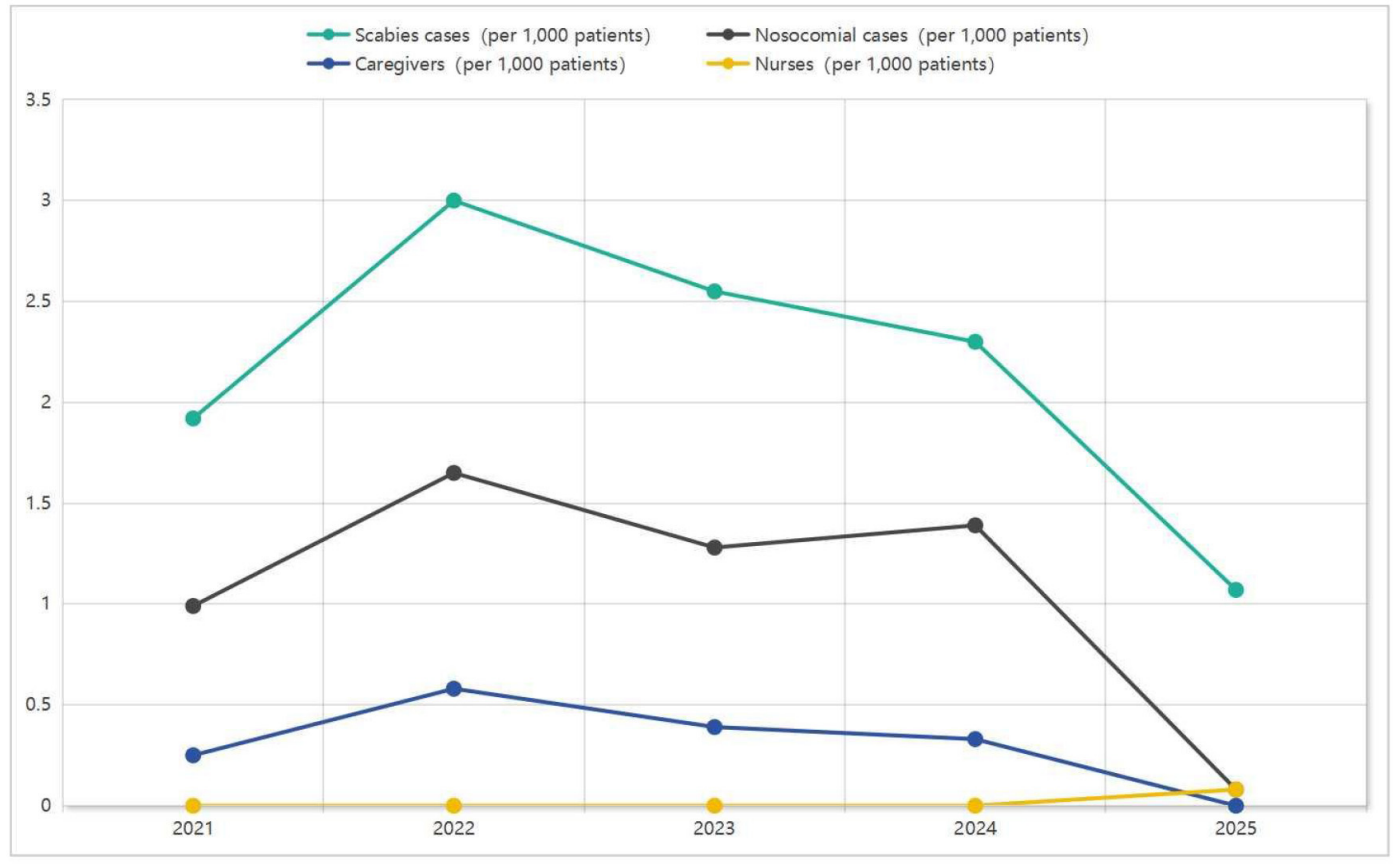
Temporal trends in annual incidence proportion (risk) (per 1,000 patients), 2021–2025.

Following the implementation of a set of comprehensive intervention strategies in 2025, 13 imported scabies cases (1.07 per 1,000 patients) were identified and promptly isolated via mandatory admission screening. Throughout the one-year surveillance period post-intervention, no caregivers infections were reported, although a single instance of nosocomial transmission involving nurse was recorded (0.08 per 1,000 patients). As illustrated in Table 1 and Figure 2, a comparative analysis of key metrics was conducted before and after the intervention. Statistical assessment using the chi-square (χ^2^) test revealed a statistically significant reduction in the incidence of nosocomial scabies transmission events following the intervention (*P* < 0.001).

**Table 1:** Comparison of scabies cases and transmission events before and after the implementation of the prevention bundle.

Year	Pre-intervention period					Post-intervention period	χ^2^	*p*
	2021	2022	2023	2024	Average	2025		
Total patients	12,009	10,333	12,544	12,150	11,759	12,175		
Scabies cases (per 1,000 patients)	1.92	3.00	2.55	2.30	2.42	1.07	4.95	0.02
Nosocomial cases (per 1,000 patients)	1.00	1.65	1.28	1.40	1.32	0.08	8.12	0.004
Caregivers (per 1,000 patients)	0.25	0.58	0.40	0.33	0.38	0		
Nurses (per 1,000 patients)	0	0	0	0.00	0	0.08		

Data are presented as *n*. Pre-intervention averages are arithmetic means. χ^2^ test was used to compare the annual number of scabies cases and nosocomial transmission events between the pre-intervention (2021–2024 aggregated) and post-intervention (2025) periods. A comparison for caregiver infections was not performed due to zero events in the post-intervention period.

### Nosocomial transmission event description

On November 14, 2025, an intensive care unit nurse presented with pruritic skin rash on the upper extremities. Subsequent dermatological consultation confirmed the diagnosis of crusted scabies. Epidemiological investigation revealed that the nurse had been providing direct care for a patient transferred from a nursing home over the preceding three days. The transferred patient was in critical condition, presenting with generalized xeroderma as well as severe cutaneous lesions characterized by hyperkeratosis and crust formation in the neck and trunk flexures. Upon admission, the patient had been initially diagnosed with atopic dermatitis following a dermatological assessment. After the nurse's diagnosis of scabies, a repeat skin scraping and microscopic examination was performed on the patient, which detected scabies mites and ova, thereby confirming the diagnosis of scabies. The patient was promptly placed under isolation for targeted treatment, and terminal disinfection of the ward was immediately implemented.

## Discussion

The prevalence of scabies in a healthcare setting could be underreported, often due to diagnostic delay and the presence of older adult(s) immunocompromised in acute or long-term care facilities, with significant economic implications related to pharmaceutical costs, lost working days, or closures of hospital wards (4, 8–10). This before-after study demonstrates that implementing a proactive, multimodal prevention bundle was associated with a dramatic and statistically significant reduction in nosocomial scabies transmission in a high-risk geriatric hospital setting, where reactive, case-based isolation had repeatedly failed. Our strategy successfully intercepted imported cases at admission and nearly eliminated in-hospital transmission chains, supporting a paradigm shift from passive containment to active prevention.

Recurrent outbreaks in our facility prior to 2025 highlighted the inadequacy of isolating identified cases alone. Diagnostic delays, atypical presentations in the older adult(s), and the historical practice of caregivers attending multiple patients created persistent reservoirs and transmission pathways.

Our bundled approach aligns with and extends key principles identified in outbreak management literature: the critical importance of early case detection, stringent contact precautions, and systematic management of healthcare personnel (11). The success likely stems from the synergistic effect of its three core components, addressing transmission at multiple points. The bundled approach likely worked synergistically: admission screening intercepted imported cases, the “one patient, one caregiver” system disrupted cross-transmission, and weekly surveillance enabled early detection. The breakthrough case in a nurse, linked to a delayed diagnosis of crusted scabies, underscores the need for high suspicion even with negative microscopy and the importance of extending surveillance to all healthcare workers in high-risk units.

First, mandatory admission screening with transfer isolation acted as an essential firewall (12). Given that most patients are transfers from facilities with high scabies prevalence, proactive identification and immediate isolation of the 13 imported cases in 2025 prevented these individuals from becoming nuclei for ward-wide outbreaks, addressing a fundamental flaw in our previous protocol (13, 14).

Second, the management and monitoring of caregivers are the core aspects of this model (15, 16). As all the patients in our hospital are older adult(s), their personal mobility is limited or even absent, and they rely on caregivers for care. Literature indicates that caregivers, being the personnel who interact with patients most frequently and closely, are both victims and important amplifiers of the spread during the epidemic (17). The “one patient, one caregiver” model in our hospital's history was a fatal weakness that led to cross-infection. Implementing the mandatory “one patient, one caregiver” system is equivalent to cutting off the main path through which scabies mites move between different patients via the caregivers” hands, work clothes, or bodies (18, 19). Combined with weekly active monitoring, it enables the early detection and removal of potential sources of infection, forming a closed loop management system.

The sole breakthrough infection in a nurse was traced to a delayed diagnosis in the index patient, whose atypical presentation (crusted scabies masquerading as dermatitis) evaded initial screening. This underscores the diagnostic challenge posed by crusted scabies in debilitated patients and indicates a vulnerability in our bundle when faced with atypical index cases. And due to the special nature of the ICU being constantly at a constant temperature, nurses wearing short-sleeved work clothes increased their exposure opportunities. According to the scabies diagnostic criteria of the International Scabies Control Alliance, the diagnosis of scabies mainly relies on microscopic examination, contact history, and itching symptoms (20). Crusted scabies is more likely to occur in patients with tumors, low immune function, malnutrition, and long-term bed rest (21). General scabies is rarely transmitted indirectly through daily items. However, the crust-forming type of scabies can be transmitted through brief contact or exposure to objects used by the patient or their skin flakes. Moreover, the sensitivity of the microscopic examination method is low, and in clinical practice, one cannot rely on a negative result from this method to rule out the presence of scabies patients (22, 23). Among these patients, those with cognitive impairments cannot complain of itching symptoms, or previous hormone treatment has changed the symptoms and signs of the patients, which can easily lead to misdiagnosis (24). This case also highlights the diagnostic difficulty of crusted scabies in older adult(s), bedridden patients, who may present with atypical features such as generalized xerosis and hyperkeratosis without classic burrows or itching, leading to misdiagnosis as dermatitis. Clinicians should maintain a low threshold for repeated skin scrapings and empirical treatment in suspicious cases, especially when there is epidemiological linkage. Since the close patient care in our hospital is mostly undertaken by caregivers, in the previous strategy, we did not include nurses as the key monitoring targets. This incident highlights the need to extend active surveillance and reinforce standard precautions (including the use of long-sleeved gowns) to all healthcare workers, particularly those in high-exposure areas like ICUs, not just caregivers.

The main strengths of this study include its real-world setting, detailed intervention description, and robust pre-post comparison. However, limitations must be acknowledged. First, the single-center, before–after design is susceptible to confounding from secular trends. Second, while annual patient totals are provided, more detailed denominator data (e.g., admission or transfer dynamics) were not available. Third, the statistical comparison between aggregated pre-intervention data (2021–2024) and a single post-intervention year (2025) does not fully account for year-to-year variability; thus, results should be interpreted with caution. Fourth, the intervention was implemented as a multimodal bundle, which precludes assessment of the individual contribution of each component. Finally, a formal cost-effectiveness analysis was not conducted (5, 25, 26). Future multi-center or stepped-wedge cluster randomized studies are needed to confirm effectiveness and identify the essential elements of the bundle.

## Conclusion

In conclusion, a reactive, case-based approach is insufficient to prevent recurrent scabies outbreaks in congregate geriatric care settings. The implementation of a proactive bundle centered on universal admission screening, a dedicated “one patient, one caregiver” model with active health worker surveillance, and multidisciplinary oversight proved highly effective. We recommend integrating such proactive, multimodal strategies into the standard infection control protocols of long-term care facilities and geriatric hospitals to sustainably break the cycle of nosocomial scabies transmission.

## Data Availability

The original contributions presented in the study are included in the article/supplementary material, further inquiries can be directed to the corresponding author.
