# Correction: Controlling recurrent scabies outbreaks in a geriatric hospital: impact of a prevention bundle and lessons from a breakthrough case

**DOI:** 10.3389/fpubh.2026.1849130

**Published:** 2026-04-17

**Authors:** Lanchuan Li, Renhua Li, Yifei Wang, Keli Qian

**Affiliations:** 1 Department of Infection Control, The Thirteenth People's Hospital of Chongqing, Chongqing, China; 2 Department of Infection Control, The First Affiliated Hospital of Chongqing Medical University, Chongqing, China

**Keywords:** caregiver management, comprehensive intervention, geriatric hospital, nosocomial transmission, scabies

Authors Lanchuan Li and Renhua Li were erroneously assigned as equal contributing authors.

Author Yifei Wang was erroneously omitted as one of the co-corresponding authors.

The original version of this article has been updated.

